# Heterogenic transplantation of bone marrow-derived rhesus macaque mesenchymal stem cells ameliorates liver fibrosis induced by carbon tetrachloride in mouse

**DOI:** 10.7717/peerj.4336

**Published:** 2018-02-12

**Authors:** Xufeng Fu, Bin Jiang, Bingrong Zheng, Yaping Yan, Junfeng Wang, Yanchao Duan, Shanshan Li, Li Yan, Hong Wang, Bingbing Chen, Xiongbo Sang, Weizhi Ji, Ren-He Xu, Wei Si

**Affiliations:** 1 Yunnan Key Laboratory of Primate Biomedical Research, Institute of Primate Translational Medicine, Kunming University of Science and Technology, Kunming, Yunnan, China; 2 Faculty of Health Sciences, University of Macau, Taipa, Macau; 3 School of Medicine, Yunnan University, Kunming, Yunnan, China; 4 Department of Hepatic and Bile Duct Surgery, The First People’s Hospital of Yunnan Province, Kunming, Yunnan, China; 5 Key Laboratory of Fertility Preservation and Maintenance of Ministry of Education, Ningxia Medical University, Yinchuan, Ningxia, China; 6 Yunnan Provincial Academy of Science and Technology, Kunming, Yunnan, China

**Keywords:** Mesenchymal stem cells, Liver fibrosis, Paracrine effects, Rhesus macaque

## Abstract

Liver fibrosis is a disease that causes high morbidity and has become a major health problem. Liver fibrosis can lead to the end stage of liver diseases (livercirrhosisand hepatocellularcarcinoma). Currently, liver transplantation is the only effective treatment for end-stage liver disease. However, the shortage of organ donors, high cost of medical surgery, immunological rejection and transplantation complications severely hamper liver transplantation therapy. Mesenchymal stem cells (MSCs) have been regarded as promising cells for clinical applications in stem cell therapy in the treatment of liver diseases due to their unique multipotent differentiation capacity, immunoregulation and paracrine effects. Although liver fibrosis improvements by MSC transplantation in preclinical experiments as well as clinical trials have been reported, the *in vivo* fate of MSCs after transportation and their therapeutic mechanisms remain unclear. In this present study, we isolated MSCs from the bone marrow of rhesus macaques. The cells exhibited typical MSC markers and could differentiate into chondrocytes, osteocytes, and adipocytes, which were not affected by labeling with enhanced green fluorescent protein (EGFP). The harvested MSCs respond to interferon-γ stimulation and have the ability to inhibit lymphocyte proliferation* in vitro*. EGFP-labeled MSCs (1 × 10^6^ cells) were transplanted into mice with carbon tetrachloride-induced liver fibrosis via tail vein injection. The ability of the heterogenic MSC infusion to ameliorate liver fibrosis in mice was evaluated by a blood plasma chemistry index, pathological examination and liver fibrosis-associated gene expression. Additionally, a small number of MSCs that homed and engrafted in the mouse liver tissues were evaluated by immunofluorescence analysis. Our results showed that the transplantation of heterogenic MSCs derived from monkey bone marrow can be used to treat liver fibrosis in the mouse model and that the paracrine effects of MSCs may play an important role in the improvement of liver fibrosis.

## Introduction

Liver fibrosis is a result of sustained and chronic liver injury, which is the final common pathway of chronic hepatic injury stimulated by many factors such as viral hepatitis, alcohol, drugs, metabolic diseases, and autoimmune attacks. Liver fibrosis leads to the development of liver cirrhosis and hepatocellular carcinoma at the end stage. During the process of liver fibrosis, hepatocytes undergo apoptosis, the endothelial barrier is impaired, inflammatory cells are recruited, the activation of hepatic stellate cells (HSCs) is initiated, and liver fibrosis results in an imbalance in extracellular matrix (ECM) synthesis and degradation, ultimately leading to hepatic malfunction (Ghatak et al., 2011; Malhi & Gores, 2008). Currently, liver transplantation is the only effective treatment for end-stage chronic liver injury (Fallowfield & Iredale, 2004). However, the extreme shortage of organ donors, high cost of medical surgery, immunological rejection, and transplantation complications severely hamper liver transplantation therapy. Recently, stem cell transplantations, including embryonic stem cells (ESCs), induced pluripotent stem cells (iPSCs), hematopoietic stem cells and mesenchymal stem cells, have been suggested as effective and alternative therapy for hepatic diseases (Zhang & Wang, 2013). Mesenchymal stem cells have a multipotent capacity, meaning that they can differentiate into various cell types including hepatocyte-like cells under appropriate conditions (Karim et al., 2010). In addition to tissue regeneration, the therapeutic mechanisms of MSCs also include immunosuppression, secretion of growth factors and promotion of endogenous regenerative processes. Moreover, compared to ESCs and iPSCs, MSCs cause fewer ethical issues for clinical applications and are easier to obtain and culture with a low risk of tumorigenesis (Alviano et al., 2007; Kamm, 2005). Therefore, these properties make MSCs one of the promising adult stem cells for clinical applications in cell therapy and regenerative medicine and can be applied in the treatment of a variety of clinical conditions including liver diseases.

Many trophic factors secreted by MSCs can lead to the survival of hepatocytes via anti-apoptotic, mitogenic, and angiogenic effects, such as stromal cell-derived factor 1(SDF-1), hepatocyte growth factor (HGF), insulin-like growth factor 1 (IGF-1), vascular endothelial growth factor (VEGF), epidermal growth factor (EGF), nerve growth factor (NGF) and transforming growth factor α (TGF-α) (Kim et al., 2012; Wang et al., 2012a). These trophic factors can reduce the proliferation of activated HSCs and collagen synthesis via the secretion of HGF and TGF-α. In addition, the generation of reactive oxygen species (ROS) that trigger oxidative stress and induce liver fibrosis can be depressed by MSCs, and liver injury can be reduced accordingly through anti-oxidant activities *in vivo* (Cho et al., 2012; Tanikawa & Torimura, 2006).

Bone marrow-derived MSCs can be easily harvested from the bone marrow and expanded *in vitro* as well as differentiated into many cell type lineages. Due to their immunotolerant superiority, MSCs have been applied in heterogenic and allogeneic transplantations in animal models for the treatment of liver diseases (Götherström, 2011; Shi et al., 2016; Zhang, Jiang & Miao, 2011). Although previous studies have reported the positive therapeutic potency of MSCs for the treatment of liver fibrosis, the mechanism of MSCs such as whether transplanted MSCs ameliorate liver fibrosis via differentiation into hepatocytes to substitute for aberrant cells *in situ* or via secretion of cytokines to promote hepatocyte regeneration *in vivo* remains unclear. Regarding this controversy, in the present study, EGFP-labeled bone marrow-derived MSCs from rhesus macaques labeled with enhanced green fluorescent protein (EGFP) were infused into mice with carbon tetrachloride-induced liver fibrosis. The efficiency of MSCs in the liver fibrosis treatment was evaluated, and the quantity and status of rhesus MSCs engrafted into mouse liver tissues were examined.

## Materials and Methods

### Animals

Mature female 4-week-old Kunming mice (Charles River, Beijing, China) with a body weight between 30 and 40 g were used for the carbon tetrachloride-induced liver fibrosis mouse model. Mice were housed in a room with a 12 h light:12 h dark cycle and provided with sterile food and water *ad libitum*. The temperature was controlled at 22 °C. Three male rhesus macaques (two years old) were used as bone marrow donors. The procedures for the monkey bone marrow retrieval, mouse model generation and cell transplantation were approved by the Institutional Animal Care and Use Committee of Kunming University of Science and Technology and were performed in accordance with the Guide for the Care and Use of Laboratory Animals. The IACUC approval number is LPBR20170201. All of the chemicals used in this study were obtained from Sigma Chemical Co. (St. Louis, MO, USA), unless otherwise indicated.

### Preparation of rhesus macaque bone marrow-derived MSCs

Bone marrow-derived MSCs were isolated from the tibias of young rhesus macaques. The muscular tissues on the tibias were carefully removed. The ends of the bones were cut, and bone marrow was aseptically flushed ten times by a sterile syringe with 10 mL of Dulbecco’s modified Eagle’s medium (DMEM) (Gibco BRL, Grand Island, NY, USA) supplemented with 10% (*v*/*v*) fetal bovine serum (FBS) (Gibco BRL, Grand Island, NY, USA) and 1% (*v*/*v*) penicillin/streptomycin (Gibco BRL, Grand Island, NY, USA). The cell suspension was then centrifuged at 500 *g* for 5 min, and the supernatant was discarded. Marrow cells were then mechanically dispersed into single-cell suspension and seeded into a 10-cm plastic dish at a density of 1 × 10^6^ cells/mL. The cells were cultured in DMEM supplemented with 10% FBS at 37 °C in an incubator with a humidified atmosphere of 5% CO_2_ in the air. The non-adherent cells were removed, and the medium was refreshed every 48 h. Ten days later, the primary cell culture (Passage 0) was passaged at 80% confluency with 0.25% trypsin (Gibco BRL, Grand Island, NY, USA). The cells were resuspended in culture medium at a dilution ratio of 1:3 and expanded on a new plastic petri dish to passage 1. The morphology, surface markers and differentiation potency of the MSCs were identified at passage 3.

### Flow cytometry analysis for surface marker profiles of MSCs

Surface marker profiles of the MSCs were examined using a commercial MSC Analysis Kit (BD Biosciences, San Jose, CA, USA) by flow cytometry analysis (BD Biosciences, San Jose, CA, USA) according to the manufacturer’s instructions. Briefly, approximately 5 × 10^5^ MSCs were collected and washed with 500 µL PBS (containing 1% FBS, PBSF). The cells were resuspended in 100 µL PBSF. Each cell sample was incubated with anti-human antibodies (Positive markers including CD44, CD90, CD73 and negative markers cocktail including CD45, CD34, CD11b, CD19, HLA-DR, which were provided by BD Co.) at a final concentration of 50 mg/L for 1 h on ice, and the isotype control antibody cocktail (mIgG1, kAPC/mIgG1, kFITC for CD73 and CD90; mIgG2b, k PE for CD44; mIgG1, k/mIgG2a, kPE for negative markers cocktail) was used as the negative control at a final concentration of 50 mg/L for 1 h on ice. Unbound antibodies were washed off with PBSF, and then the cells were re-suspended with 500 µL PBSF for flow cytometry analysis.

### Evaluation of the differentiation potential of MSCs

For adipogenic differentiation, MSCs were seeded into 24-well plates and cultured at a density of 8 × 10^4^ cells per well for 12 h. Then, the cells were cultured in an adipogenic differentiation medium (Gibco BRL, Grand Island, NY, USA) for 7 days (Ninomiya et al., 2010). The medium was refreshed every three days. The cells were stained in filtered Oil Red O (0.2% Oil Red O in 60% isopropanol, *v*/*v*) for 15 min and washed 3 times with PBS after fixation in 4% methanol. The adipogenic differentiation was confirmed by the cellular accumulation of neutral lipid vacuoles, which were stained red with Oil Red O (Sigma, St. Louis, MO, USA). For osteogenic differentiation, MSCs were seeded into 24-well plates and cultured at a density of 4 × 10^4^ cells per well for 12 h. Then, the culture medium was replaced with an osteogenic differentiation medium (Gibco BRL, Grand Island, NY, USA) and further cultured for 21 days. The medium was refreshed every three days. The cells were stained with fresh 0.5% Alizarin Red solution and washed three times with PBS after fixation with 4% methanol. The osteogenic differentiation was confirmed by the appearance of Alizarin Red stain. For chondrogenic differentiation, MSCs were collected in 15-mL centrifuge tubes at approximately 2 ×10^5^ cells per tube cultured in a chondrogenic differentiation medium (Gibco BRL, Grand Island, NY, USA). The medium was refreshed every three days. After 21 days of differentiation induction, the chondroid pellets were generated and washed with PBS, fixed in 4% paraformaldehyde, and embedded with OTC embedding material (Leica, Wetzlar, Germany). The pellets were sectioned by a freezing microtome, and then sulphated proteoglycans were visualized by staining with 1% toluidine blue (Merck, Darmstadt, Germany) for 10 min. These slices were washed three times with PBS and photographed under an inverted microscope. Differentiation was confirmed by the appearance of Alcian Blue stain.

### Cell labeling with EGFP

To track the transplanted cells *in vivo*, MSCs were labeled with EGFP by lentivirus infection. Briefly, the pLV-CMV-EGFP-Neo vector, PMD2.G and PSPAX2 packaging plasmids, and the X-tremeGENE HP DNA Transfection Reagent (Roche, Basel, Switzerland) were added to 10% FBS DMEM medium, mixed gently, and incubated at room temperature for 20 min. The mixture was added dropwise into 293T cells in a 10-cm plate. After a 48-h incubation period, the virus supernatant was collected and filtered using a 0.45- µm filter. Rhesus macaque bone marrow-derived MSCs were then infected with the virus. After being cocultured for 48 h, the aminoglycoside antibiotic G418 (Gibco-BRL, Carlsbad, CA, USA) was added to the medium at a final concentration of 600 µg/mL to select MSCs with a stable EGFP expression. The MSCs labeled with EGFP were observed with a fluorescence emission ratio at 530 nm using an epifluorescence microscope and an excitation wavelength of 488 nm, and the labeling efficiency was detected by flow cytometry analysis. The differentiation potency of adipogenic, osteogenic and chondrogenic MSCs was further examined by the methods described above after being labeled with EGFP using lentivirus infection.

### Carbon tetrachloride-induced liver fibrosis mouse model and heterogenic MSC infusion

The mice were intragastrically administered CCl_4_ (mixed with olive oil at a 1:1 volume ratio) at a dose of 0.2 mL/100 g body weight during the first week. Then, the mice were further administered CCl_4_ (mixed with olive oil at a 3:1 volume ratio) at a dose of 0.2 mL/100 g body weight three times a week for eight weeks. The mice administered with the same volume of olive oil only were used as controls. Sixty mice were randomly divided into olive oil administered mice (*n* = 10) and (2) CCl_4_ administered mice (*n* = 50). After eight weeks, several mice died from CCl_4_ toxicity, and the development of CCl_4_-induced liver fibrosis in the mice that survived was determined by the evaluation of pathological liver sections by a trained pathologist. Then, seven CCl_4_ administered mice from the 16 survivors were divided into two groups randomly, and the others were used for anther experiment, which was not involved in the present study. Three mice administered with CCl_4_ but without MSCs transplantation were used as CCl_4_ group. In contrast, four mice administered with CCl_4_ and transplanted with MSCs were used as CCl_4_+MSCs group. The EGFP-labeled rhesus MSCs at passage six were washed three times with saline and resuspended at a concentration of 5 ×10^6^ cells/mL. Then, a single dose of MSCs (1 ×10^6^ cells) was infused into the CCl_4_ administered mice via tail vein injection. Meanwhile, the same volume of saline (200 µL) was infused into the control mice. Thirty days later, each group was anesthetized using 0.3 mL/100 g of body weight of 10% chloral hydrate (Sigma, St. Louis, MO, USA) solution which was injected intraperitoneally, and the liver and venous blood were collected.

### Blood collection and plasma chemistry tests

Blood serum was separated by centrifugation at 3,000 rpm for 15 min within 1 h after blood collection and stored at −80 °C until analysis. The following plasma chemistry parameters were measured using a Roche Modular P800 automatic biochemical analyzer (Roche Diagnostics Ltd., Basel, Switzerland): aspartate aminotransferase (AST), alanine aminotransferase (ALT), albumin (ALB) and total protein (TP).

### Gene expression analyzed by qRT-PCR

Total RNA was extracted from the liver tissues of mice using Trizol Reagent (Takara, Dalian, China). The RNA was first separated into an aqueous phase by adding chloroform and then was precipitated with isopropanol, rinsed with 75% ethanol and finally solubilized in sterile DEPC water. Complementary DNA (cDNA) was synthesized using a Prime-Script RT reagent kit (Takara, Dalian, China) according to the manufacturer’s recommendations. Highly purified gene-specific primers are listed in Table 1, including the housekeeping gene glyceraldehyde-3-phosphate dehydrogenase (GAPDH), α-smooth muscle actin (α-SMA), albumin (ALB), tumor necrosis factor (TNF-β) and alpha fetal protein (AFP), which were commercially synthesized (Shengong, Shanghai, China). Quantification of the cDNA of the specific genes was performed with a Bio-Rad CXF real-time PCR system. All experiments were performed in triplicate, and the data were analyzed by the 2^−△Ct^ procedure.

**Table 1:** Primers for RT-PCR.

Gene name	Species	Forward primer	Reverse primer
GAPDH	*Maus*	ACGGATTTGGTCGTATTGG	GCTCCTGGAAGATGGTGAT
α-SMA	*Maus*	GGGAGTAATGGTTGGAATGG	ACAGCACAGCCTGAATAGCC
ALB	*Maus*	ATACACCCAGAAAGCACCTC	GCTGTAGCCTTGGGCTTG
TNF-β	*Maus*	AACTCGAGTGACAAGCCCGTAG	GTACCACCAGTTGGTTGTCTTTGA
AFP	*Maus*	CAGTGCGTGACGGAGAAGA	CTAAACACCCATCGCCAGAG
EGFP	*Medusa*	TCGTGACCACCCTGACCTA	CACCTTGATGCCGTTCTTCT
GAPDH	*Macaca*	TGTTGCCATCAATGACCCCT	ATGACGAGCTTCCCGTTCTC
IOD1	*Macaca*	TGCACGATCACGTAAACCCA	ATAGCTGGGGGTTGCCTTTC
CCL2	*Macaca*	AGCCAGATGCAATCAATGCC	GGGTCAGCACAGATCTCCTT
CXCL10	*Macaca*	GTAAGCTGTACTGCGGTGCT	AGGGAAATACCGGAAGCAGG
CXCR4	*Macaca*	ATCAGTCTGGACCGCTACCT	CCACCTTTTCAGCCAACAGC

### Histological and immunofluorescence analysis

Liver tissue samples were collected from the euthanized mice and fixed in 4% formaldehyde for two days. Tissues were then dehydrated, cleared and infiltrated with a histoprocessor for 16 h. The liver tissues were sectioned into 3-µm pieces and stained with hematoxylin and eosin (H&E) or Masson’s Trichrome solutions. For H&E analysis, sectioned samples were stained with hematoxylin solution (Sigma-Aldrich, Munich, Germany) for 5 min, followed by eosin for 5 min. For Masson’s trichrome stain, sectioned samples were placed in Bouin’s solution at 56 °C for 1 h and stained in succession with Mayer’s hematoxylin solution, Biebrich Scarlet-acid fuchsin solution, phosphomolybdic acid–phosphotungstic acid and aniline blue for 5 min, 15 min, 15 min and 5 min, respectively. All of the staining reagents were from Sigma-Aldrich (St. Louis, MO, USA). Specimens were evaluated with respect to inflammation, ballooning degeneration, and collagen deposition by an experienced veterinary pathologist who was blinded to the treatment that the animals received.

For immunofluorescence, liver tissues were cut into cubes of about 1 cm ×1 cm in size, fixed in 4% paraformaldehyde for 48 h, and dehydrated in 15%, 20% and 30% sucrose solution for 12 h. Then, the liver tissue samples were embedded in OTC embedding material (Leica, Wetzlar, Germany) and sectioned into 8-µm sections, which were incubated with 0.1% Triton in PBS for 30 min. After being washed with PBS, the slices were incubated with the blocking solution (PBS containing 10% goat serum), incubated overnight with primary antibodies (EGFP, Ki-67, and caspase-3 purchased from Abcam, Cambridge, UK), and finally incubated with rabbit secondary antibodies (conjugated with FITC and PE, purchased from Abcam, Cambridge, UK) at 37 °C for 2 h after being washed with PBS. All matched samples were photographed using an immunofluorescence microscope. Immunofluorescence images were analyzed using Image J (NIH, Bethesda, MD, USA) after being stained with DAPI (4′,6-dia-midino-2-phenylindole).

### DNA extraction and PCR product detection

Genomic DNA was extracted from the liver tissue using DNA extraction kits (Tiangen, Beijing, China) following the manufacturer’s instructions. Genomic DNA extracted from the EGFP-labeled MSCs was used as a positive control. The concentration of the extracted DNA was measured by a NanoDrop 2000 (Thermo Fisher Scientific, Waltham, MA, USA). For PCR, the reaction mixture contained 20 ng of DNA template, 1 µL of EGFP primers (forward and reverse primers at a concentration of 10 µM, primers are listed in Table 1), 10 µL of 2 × Taq PCR Mix (Dingguo, Beijing, China), and ddH_2_O of up to 20 µL. The PCR reaction was conducted for 30 cycles. Each cycle consisted of the following steps: 94 °C for 2 min, 94 °C for 40 s, 66 °C for 30 s, and 72 °C for 150 s. After 30 cycles, the samples were subjected to a final step at 72 °C for 10 min. PCR products were separated by agarose gel electrophoresis.

### Inhibition of T lymphocyte proliferation *in vitro*

Lymphocytes derived from the lymph nodes of 10 week-old Kunming mice were first labeled with 5 µM CFDA SE Cell Proliferation Assay and Tracking Kit (Beyotime, Jiangsu, China) for 10 min in a 37 °C water bath and then cultured in 1640 complete medium (RPMI-1640 plus 10% FBS) in a 96-well plate at a density of 2 ×10^5^ cells/100 µL/well. Rhesus MSCs were expanded in a 6-well plate and cultured for five days. Then, the MSCs were dissociated with 0.05% Trypsin-EDTA and added to the 96-well plate cultured with lymphocytes. The ratio of MSCs to lymphocytes was 1:40 or 1:80 per well. The lymphocytes in the mixture were stimulated with or without pre-coated 10-µg/ml anti-CD3 antibodies (BD Biosciences, San Jose, CA, USA) and 2 µg/mL anti-CD28 antibodies (BD Biosciences, San Jose, CA, USA) or on beads. Lymphocytes were collected 5 days later after stimulation and then subjected to flow cytometry analysis. The data were analyzed with Flowjo (TreeStar, Ashland, OR, USA).

### Response of rhesus macaque bone marrow-derived MSCs to IFNγ and reverse transcription-polymerase chain reaction (RT-PCR)

A total of 10^5^ MSCs were loaded in a 6-well plate and treated with 20 ng/mL IFNγ for 24 h. Total RNA was extracted from the cultured MSCs using Trizol reagent (Life Technology, Carlsbad, CA, USA), and cDNA was generated using the PrimeScript RT Reagent kit (Clontech, Mountain View, CA, USA). PCR reactions were carried out using the Quick-Load^®^ Taq 2 × Master Mix (NEB). GADPH, indoleamine 2,3-dioxygenase 1 (IDO1), C-C motif chemokine ligand 2 (CCL2), chemokine (C-X-C motif) ligand 10 (CXCL10) and C-X-C motif chemokine receptor 4 (CXCR4) primers were commercially synthesized, and the sequences are listed in Table 1. The PCR conditions are as follows: an initial denaturation at 95 °C for 25 s, followed by 28–32 cycles of 25 s, denaturation at 95 °C for 20 s, annealing at 56 °C for 25 s, extension at 68 °C, and a final extension at 68 °C for 5 min. The PCR product was analyzed by electrophoresis in a 1.5% gel.

### Data analysis

All of the data are expressed as the means ± SEM. The statistical differences were analyzed using Student’s *t*-test, and a *P* value less than 0.05 was considered significantly different. Histograms were drawn with GraphPad Prism 5 (GraphPad Software, San Diego, CA, USA).

## Results

### Morphology, surface marker profiles and differentiation potency of MSCs

**Figure 1:**
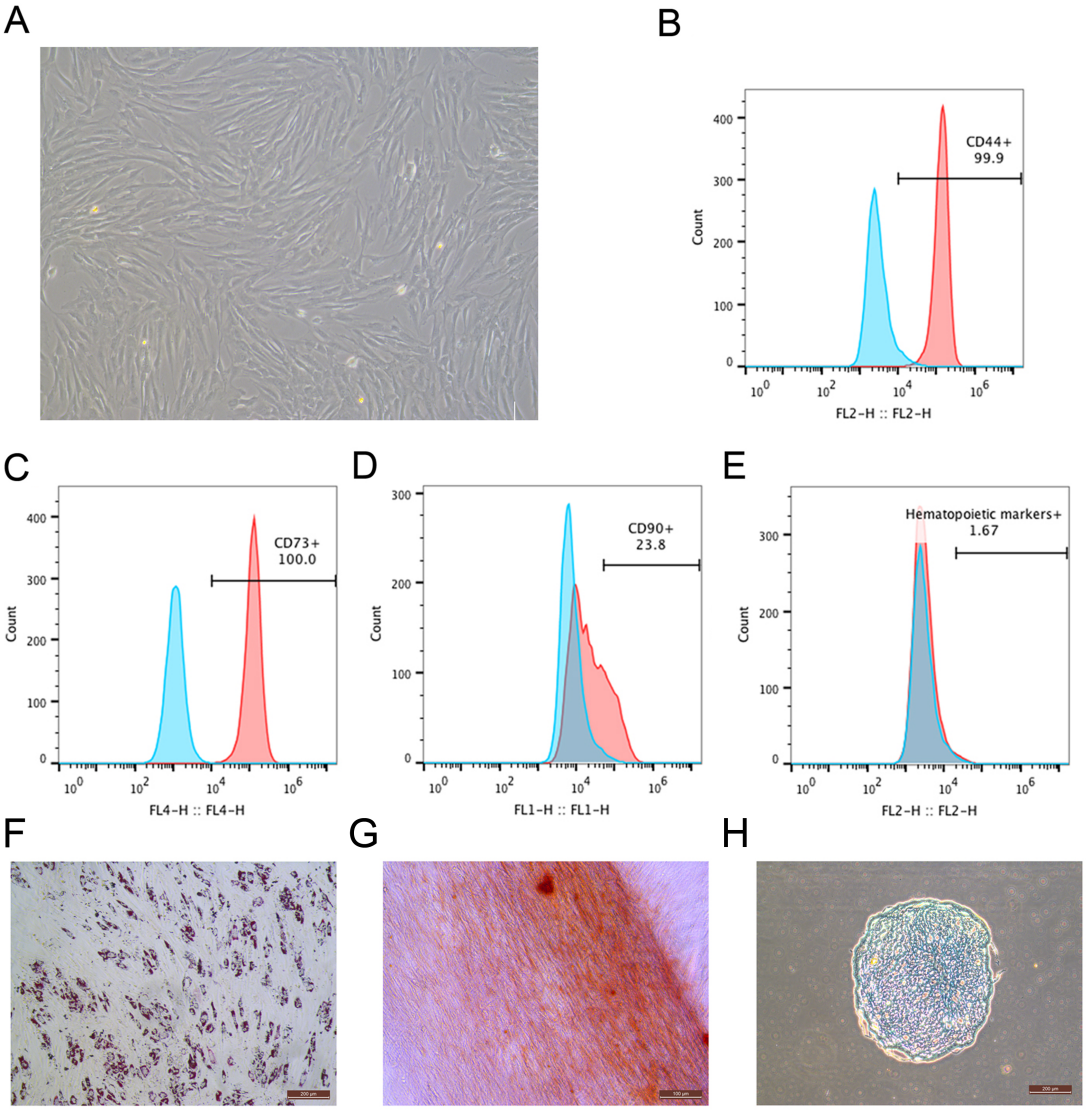
Identification of rhesus macaque bone marrow-derived MSCs. (A) The fibroblast-like morphology of rhesus macaque bone marrow-derived MSCs at passage 3; (B–E) Surface markers expression on the bone marrow-derived MSCs analyzed by flow cytometry, (B) CD44; (C) CD73; (D) CD90; (E) Hematopoietic markers; Blue peak diagram represents the isotype control; (F) Adipogenic differentiation (Oil Red O staining, ×200); (G) Osteogenic differentiation (Alizarin red staining, ×200); (H) Chondrogenic differentiation (Alcian Blue staining, ×200). Scale bars: (A) and (F–H) are 200 µm.

During the primary culture, the MSCs derived from macaque bone marrow adhered to the plastic dishes in a scattered manner. The MSCs appeared like typical homogeneous fibroblast-like, elongated, spindle-shaped, heterogeneous, with single nucleus features following subsequent culture (Fig. 1A). The surface marker profiles of the MSCs were analyzed by flow cytometry. The results indicated that the cells positively expressed high levels of CD44 and CD73 but negatively expressed hematopoietic markers (Figs. 1B–1E). The bone marrow-derived MSCs could differentiate into adipocytes (Fig. 1F), osteocytes (Fig. 1G) and chondrocytes (Fig. 1H). After being submitted to an adipogenic induction, numerous neutral lipid droplets stained by Oil Red were observed in the cytoplasm of cells. After being submitted to an osteogenic induction, the cells presented an aggregation of micronodules or calcium deposits that were stained by Alizarin Red. The chondrogenic differentiation of MSCs could be observed using an Alcian Blue stain.

### Efficiency of MSCs labeled with EGFP and potency of labeled MSCs

**Figure 2:**
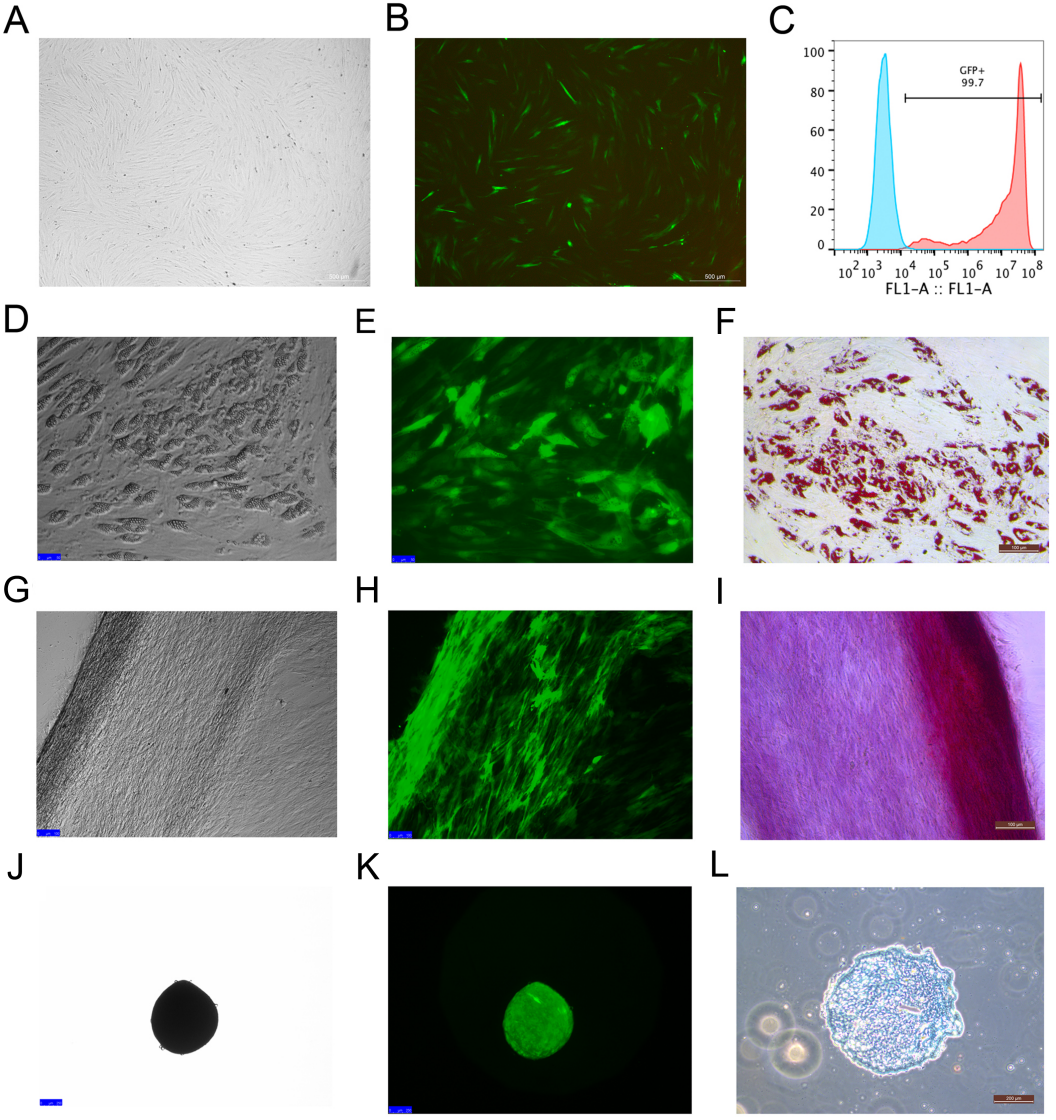
MSCs labeled with EGFP by lentivirus infection and the differentiation potency of the EGFP-positive MSCs. (A) MSCs labeled with EGFP in light field; (B) MSCs labeled with EGFPin fluorescence field. (C) EGFP labeling efficiency as detected by flow cytometry. The blue line on the left side indicates the isotype control, and the red line on the right side indicates the EGFP-labeled MSCs. (D) Adipogenic differentiation, (G) Osteogenic differentiation and (J) Chondrogenic differentiation of the MSCs labeled with EGFP under a light microscope; (E) Adipogenic differentiation, (H) Osteogenic differentiation and (K) Chondrogenic differentiation of the MSCs labeled with EGFP under a fluorescence microscope; (F) Adipogenic differentiation (Oil Red O staining), (I) Osteogenic differentiation (Alizarin red staining) and (L) Chondrogenic differentiation (Alcian Blue staining) of the MSCs labeled with EGFP under a light microscope. Scale bars: (A), (B), (D–I) and (L) are 200 µm, (J) and (K) are 100 µm.

Rhesus macaque bone marrow-derived MSCs were infected by lentiviruses, which were packaged using 293T cells. After 48 h of infection, the MSCs that expressed EGFP emitted an obviously green fluorescence that was observed under a fluorescence microscope (Figs. 2A and 2B). The efficiency of the MSCs labeled with EGFP was 99 ± 0.6% as detected by flow cytometry analysis (Fig. 2C), which indicated that EGFP labeling was successful with high efficiency through the lentivirus infection method. The MSCs labeled with EGFP had the capacity to differentiate into adipocytes (Figs. 2D–2F), osteocytes (Figs. 2G–2I) and chondrocytes (Figs. 2J–2L), which were identified by Oil Red O, Alizarin Red and Alcian Blue staining, respectively.

### Generation of the liver fibrosis mouse model and EGFP-labeled MSC therapy

**Figure 3:**
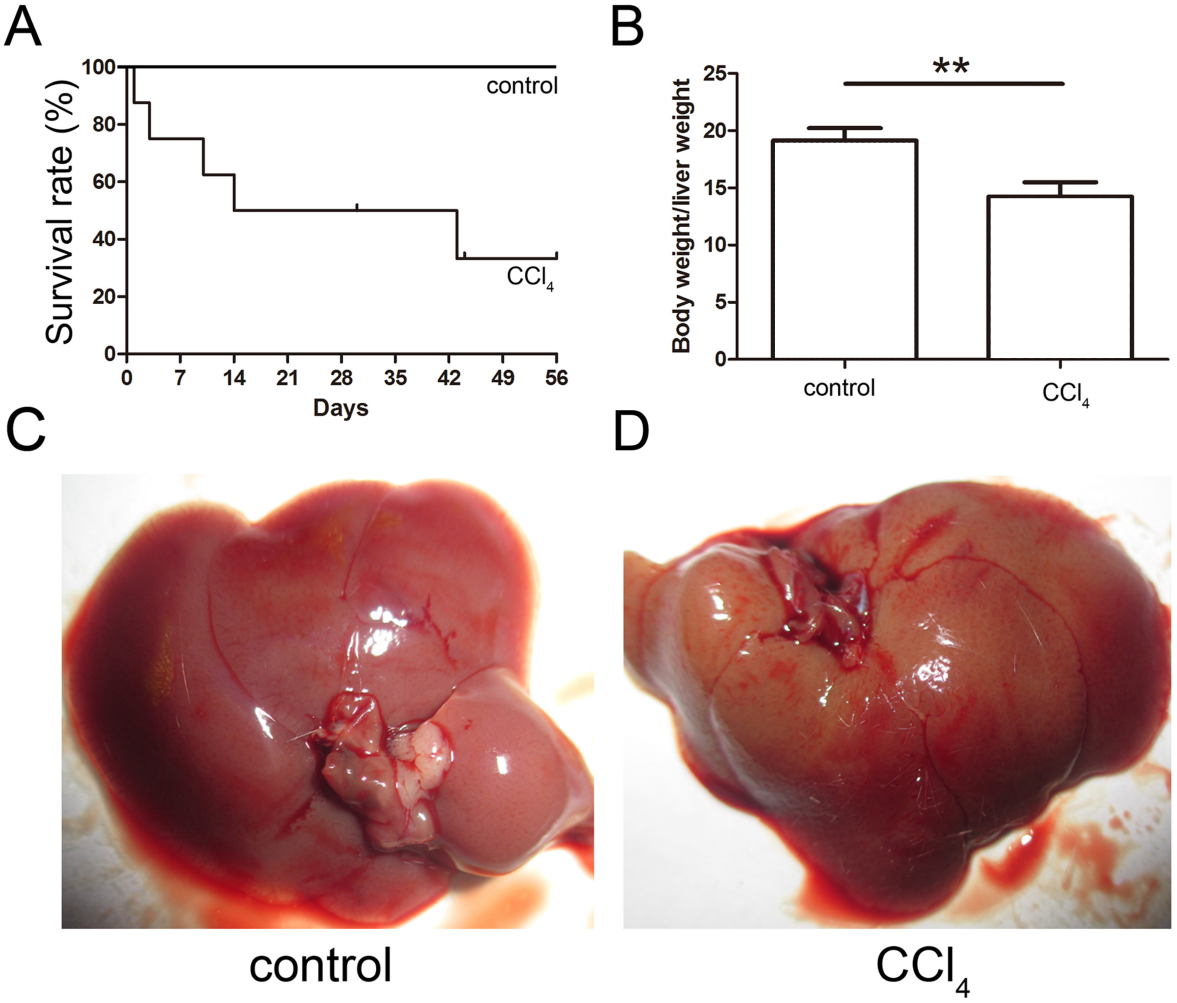
Generation of the liver fibrosis mouse model. (A) Survival rate of the CCl_4_-induced mouse model. (B) The ratio of the body weight and liver weight in the control and model groups. (C) and (D) Morphological comparison of livers between the control and CCl_4_-administered model groups.

The toxicity of CCl_4_ caused 68% of the mice to die after eight weeks of administration, and the final survival rate was 32% as shown in Fig. 3A. The ratio of the body weight to the liver weight of CCl_4_-administered mice was lower than that of the control group (Fig. 3B). The livers of both the control and CCl_4_-administered groups were collected after necropsy, and the CCl_4_-administered liver exhibited enlargement, an ashen appearance and a messy irregular surface structure compared to the normal liver, which was pink or purplish-red and smooth as shown in Figs. 3C and 3D.

**Figure 4:**
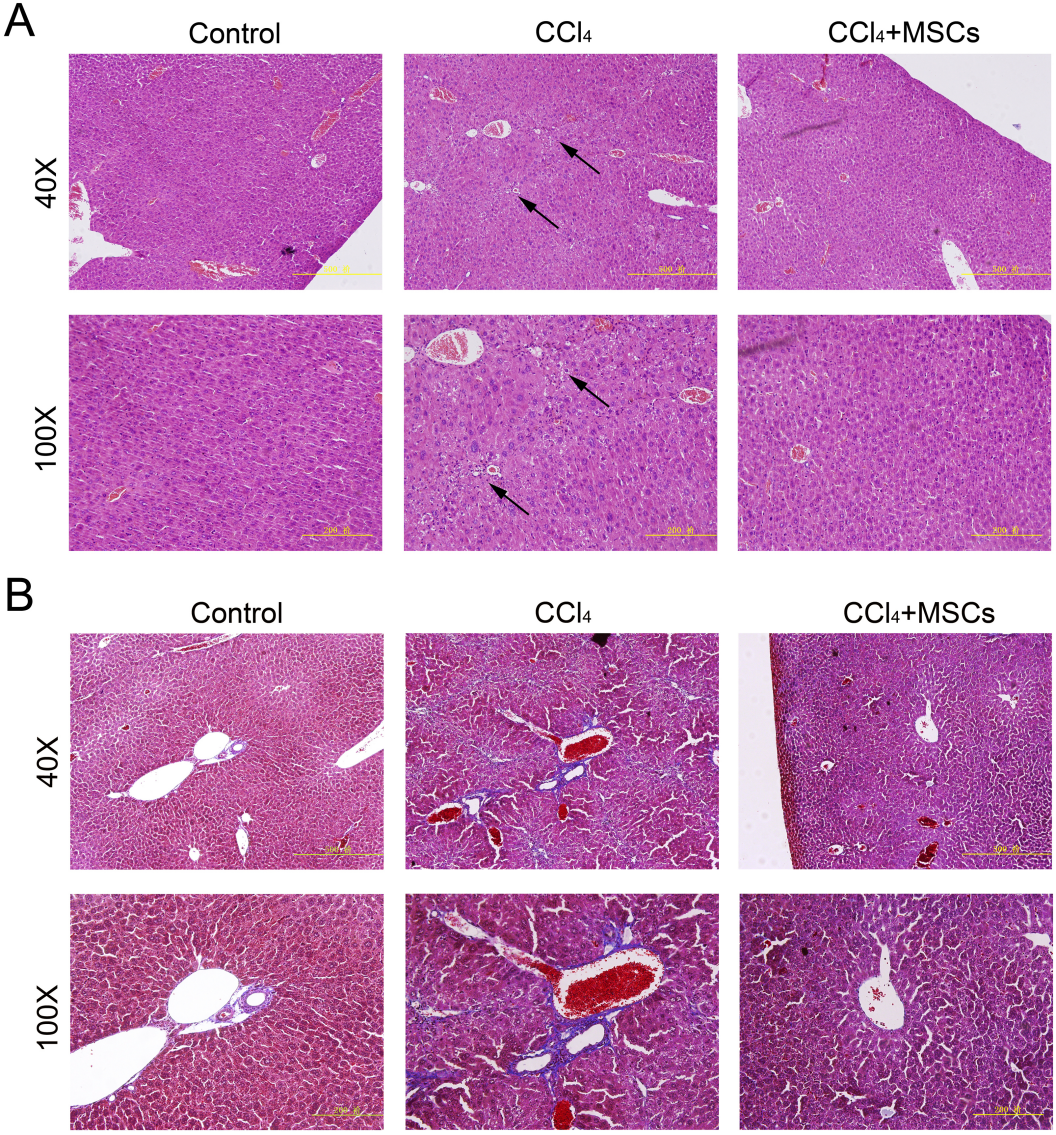
Heterogenic transplantation of MSCs ameliorates liver fibrosis in mice. Significantly decreased hepatocyte necrosis (A) and collagenous fibers around the blood vessel (B), which was confirmed by H&E and Masson’s trichrome staining after EGFP-labeled MSCs delivery. Three normal mice were selected as control group without CCl_4_ administration and MSCs transplantation, three mice administrated with CCl_4_ but without MSCs transplantation were used as CCl_4_ group and four mice were transplanted with MSCs as the cell therapy group. Arrows in (A) represent the sites of hepatocyte necrosis.

**Figure 5:**
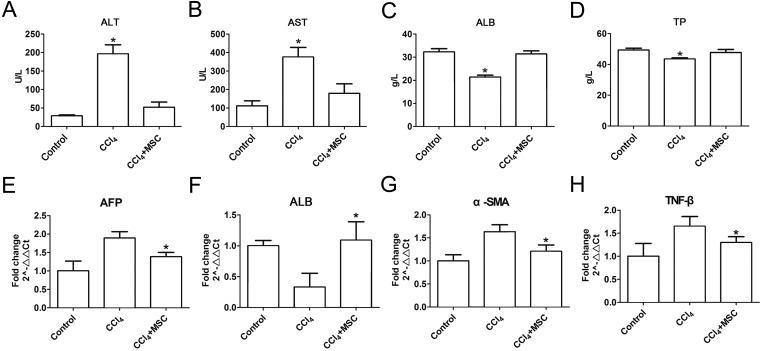
Heterogenic MSCs ameliorates liver function and decreases fibrosis liver fibrosis molecules. (A–D) Liver function markers, (A) ALT: Alanine aminotransferase; (B) AST: Aspartate amino transferase; (C) ALB: albumin; (D) TP: Total protein. (E–H) Iconic liver fibrosis molecules, (E) AFP: Alpha fetoprotein; (F) ALB: albumin; (G) α-SMA: α-smooth muscle actin; (H) TNF-β: Tumor necrosis factor β. These data are representative of four animals.

Consequently, EGFP-labeled MSCs were transplanted via the tail vein. Four weeks later, the results of the pathological evaluation, serum biochemical index of the liver function and liver fibrosis-related gene expression were discovered, which are shown in Fig. 4. Compared to the control, the histological examination of H&E and Masson stained liver sections of the CCl_4_-administered mice revealed the development of necroinflammatory changes and densely deposited collagen fibers. In contrast, the inflammation and ballooning degeneration in liver tissue were obviously alleviated four weeks after MSC infusion (Figs. 4A and 4B). Blood serum ALT, AST, ALB and TP concentrations of the CCl_4_-administered mice with liver fibrosis were elevated compared to the control group, and the level of these indexes decreased to the normal level four weeks after the infusion of MSCs as shown in Figs. 5A–5D. The mNA levels of ALB, α-SMA, AFP and TNF-β in the CCl_4_-administered mice with liver fibrosis were significantly increased compared to the control group, and the mRNA level of these genes increased to the normal level four weeks after MSCs infusion, as shown in Figs. 5E–5H.

### The status of EGFP-labeled MSCs homed into liver tissue of CCl_4_- administered mice with liver fibrosis

**Figure 6:**
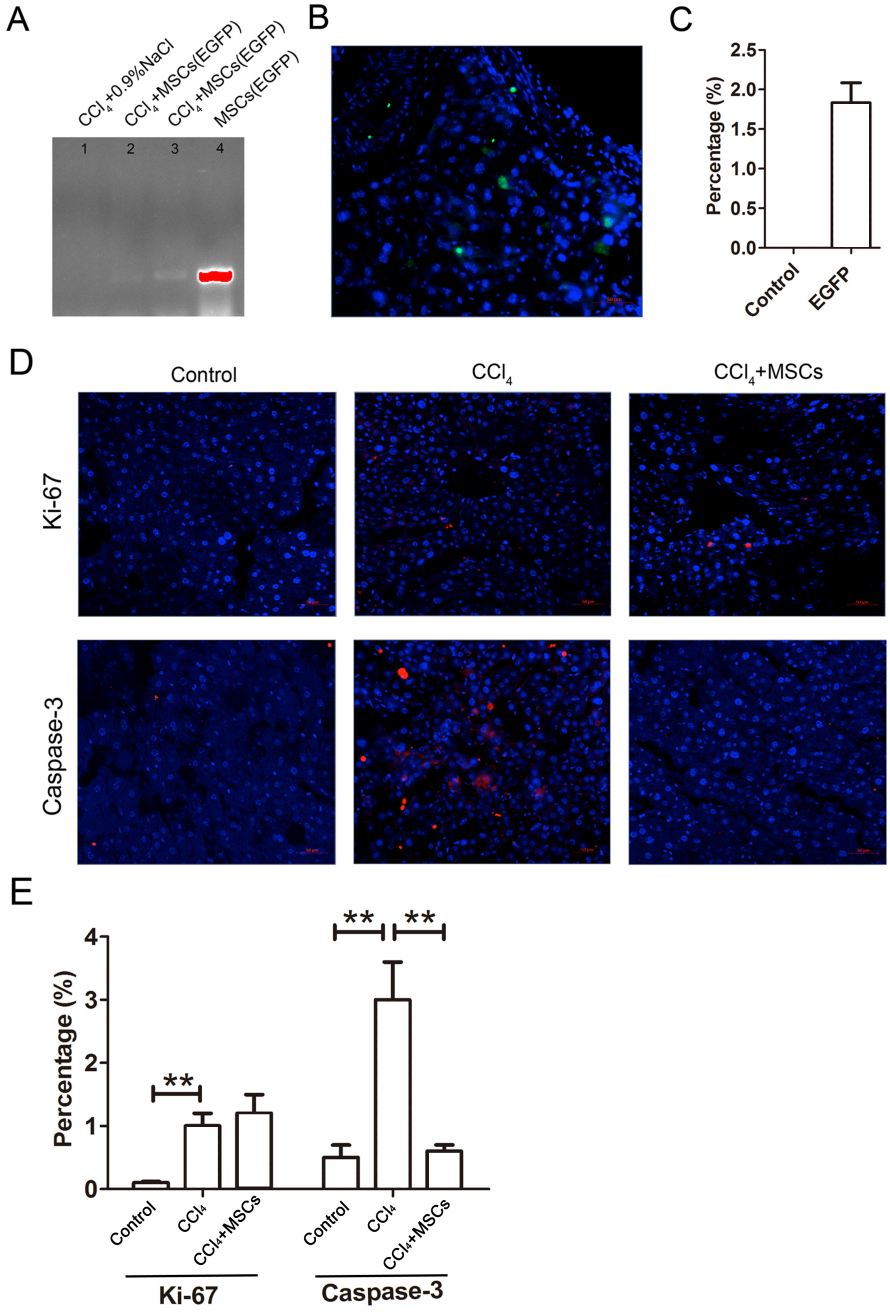
EGFP-labeled MSCs homed into liver tissue of CCl_4_-administered mouse liver. (A) Weak electrophoresis bands of EGFP presented in the mouse liver tissue infused with labeled MSCs (lanes 2 and 3), where lane 1 is the negative control and lane 4 is the positive control. (B) Homed MSCs express EGFP (Green) distributed in the cells (Blue, stained with DAPI) in the mouse liver tissue under a fluorescence microscope. (C) Percentage of EGFP-labeled MSCs in the total cells from the frozen liver sections B. (D) The proteins related to proliferation (Ki-67) and apoptosis (caspase-3) were detected by immunofluorescence (Red). (E) Quantified percentage of cells that expressed Ki-67 and caspase-3 proteins (D). ** denotes *P* < 0.01.

The EGFP-labeled MSCs homed into the CCl_4_-administered mouse livers and were detected by PCR and immunofluorescence after four weeks of cell transplantation. In comparing the positive groups (EGFP-labeled MSCs), the result of the PCR and electrophoresis detected weak electrophoresis bands of EGFP present in the liver tissues with EGFP-labeled MSC infusions (Fig. 6A). Immunofluorescence of the frozen sections from the CCl_4_-administered mouse livers infused with EGFP-labeled MSCs showed that only 1.8 ± 0.4% of the cells in the liver tissues were MSCs (Figs. 6B and 6C). The expression levels of the proliferation and apoptosis-related hepatocyte genes (Ki-67 and caspase-3) from the frozen liver sections were compared between the control, CCl_4_-administered mice with liver fibrosis, and CCl_4_-administered mice with liver fibrosis infused with MSCs by immunofluorescence (Figs. 6D and 6E). The CCl_4_ administration significantly increased expression of the hepatic proliferation-related protein Ki-67 compared to the control, and the transplantation of MSCs did not affect the increased expression of Ki-67. In contrast, CCl_4_-administration significantly increased the expression of the hepatic apoptosis-related protein caspase-3 compared to the control, and the transplantation of MSCs alleviated the increased expression of caspase-3 to a normal level.

### MSCs have better functional characteristics

**Figure 7:**
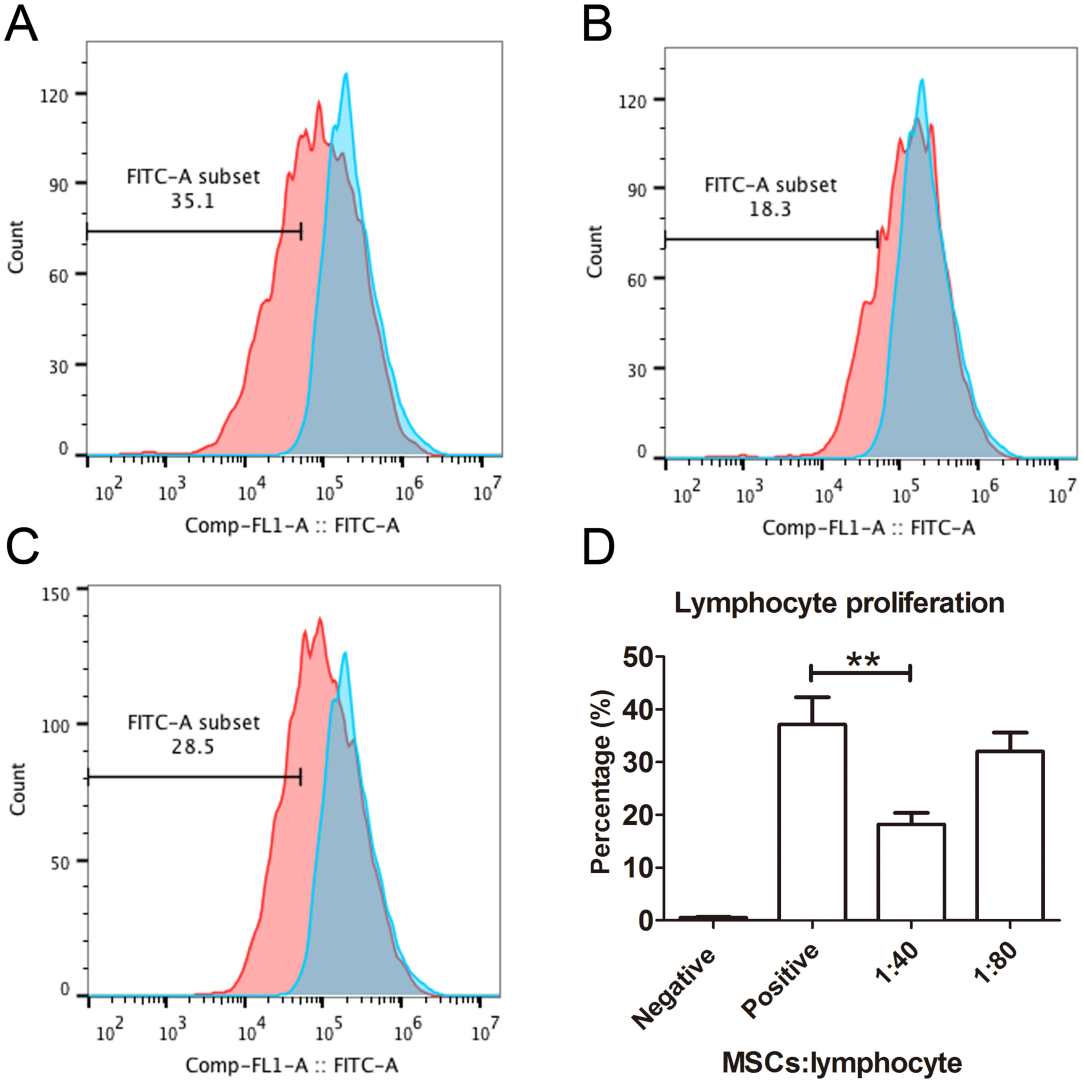
Lymphocyte proliferation is inhibited by MSCs. (A) Flow cytometry images of lymphocytes stimulated with both CD3 and CD28 antibodies as positive controls. The blue peak is the negative control with only lymphocytes and without CD3 and CD28 antibody stimulation. (B) The flow cytometry image of the lymphocytes treated with MSCs at a ratio of 1:40 (MSCs *vs* lymphocytes). (C) The flow cytometry image of the lymphocytes treated with MSCs at a ratio of 1:80. (D) The histogram of lymphocyte proliferation inhibition by MSCs.

**Figure 8:**
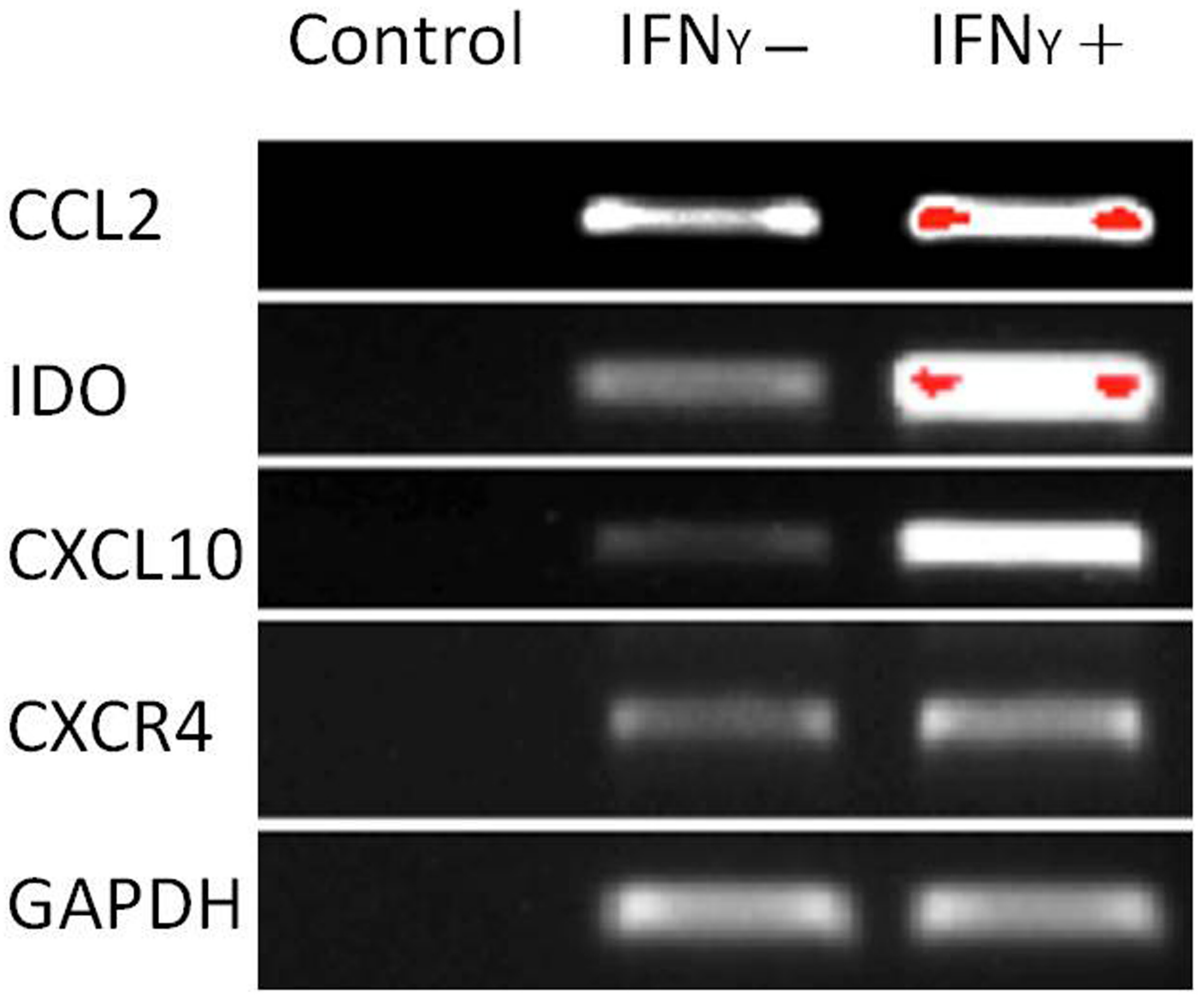
Chemokine and migration-related genes were up regulated in IFNγ-treated MSCs. MSCs were treated with 20 ng/mL IFNγ for 24 h. The chemokine and migration associated genes *CCL2, IDO1, CXCL10* and *CXCR4* were up regulated compared with the untreated siblings by RT-PCR assay. MSCs responded to inflammatory factors and potentially migrated to inflammation sites.

The capacity of MSCs to inhibit lymphocyte proliferation and secrete cytokines *in vitro* was determined by flow cytometry and qRT-PCR. The result showed that the co-culture of MSCs with lymphocytes at a ratio of 1:40 significantly decreased the proliferation rate of lymphocytes from 38.1 ± 4.4% to 18.3 ± 2.2%. However, the inhibition effect of MSCs on lymphocyte proliferation was not significant when the MSC cell ratio decreased to 1:80 (32.5 ± 3.1%) (Fig. 7). After being treated with IFNγ for 24 h, the RT-PCR results showed that the chemokine and migration-related genes, including *CCL2, IDO1, CXCL10* and *CXCR4*, in MSCs were up-regulated, which suggested that the MSCs reacted to the inflammatory factor and had the potential to migrate to inflammatory sites (Fig. 8).

## Discussion

MSCs have been regarded as promising cells for liver fibrosis due to their hepatocyte differentiation and trophic effect, which promotes hepatocyte regeneration as well as anti-fibrosis and antioxidant characteristics. Transplanted MSCs have been investigated in clinical trials as having therapeutic potential for the treatment of chronic liver disease and showed positive effects on decreased fibrosis syndrome and its related factors, the serum albumin level and the amelioration of mayo end-stage liver disease scores through peripheral vein infusion (El-Ansary et al., 2012; Kharaziha et al., 2009; Zhang et al., 2012b). Although the therapeutic potential and effectiveness of MSCs have been observed in clinical trials for the treatment of chronic liver diseases, the mechanisms of how MSCs differentiate to hepatocyte-like cells, alleviate inflammation, and secrete trophic factors at the site of liver injury is not well understood. Meanwhile, the efficiency of infused MSCs that homed and integrated into liver tissues remains unclear. Due to the invasiveness of liver tissue sampling and safety considerations of EGFP-labeled cell infusion, it is impractical to explore therapeutic mechanisms of MSCs and track the path of the migrated MSCs in the recipient’s liver in clinical trials. EGFP-labeled MSCs are good indicators for exploring the therapeutic mechanisms of stem cell transplantation in animal disease models.

In this study, MSCs were isolated from rhesus macaque bone marrow and were confirmed by morphology, surface markers, and tri-lineage differentiation. All of the results were consistent with the putative standards for MSCs by the International Society for Cellular Therapy (Dominici et al., 2006). Then, the MSCs were steadily labeled with EGFP through lentivirus infection, and the results indicated that the characteristics of MSCs including surface markers, adipogenic, osteogenic, and chondrogenic *in vitro* differentiation were not affected after transfection with lentivirus. The results were consistent with reports that the osteogenic or adipogenic differentiation potentials of the human bone marrow-derived MSCs were not affected after lentiviral vector transduction (McMahon et al., 2006; Van Damme et al., 2006). Similarly, Yu and his colleagues reported that the phenotypic profile, viability and adipogenic, osteogenic and hepatic differentiation were not affected by GFP transfection into human placenta MSCs using lentivirus infection. They also found that this GFP labeling method has no adverse effects on the cellular or biochemical properties of hMSCs in terms of the metabolic, synthetic and secretory functions of hepatocyte-like cells that differentiated from GFP-labeled hMSCs (Van Damme et al., 2006). Therefore, our results suggest that this method is practical and that labeled MSCs could be used for therapeutic transplantation due to the fact that the functions of MSCs were not affected by lentivirus transduction. The lentiviral vector transduction of the GFP gene into MSCs is useful for tracking the distribution and homing status of the infused cell.

The therapeutic effect of MSCs derived from various tissues has been reported in rodent models with liver fibrosis (Kubo et al., 2015; Yan et al., 2015; Zhu et al., 2015). Even though the dose and approach of CCl_4_ administration were different and the number of MSCs transplanted varied in these studies, it appeared that MSCs suppressed liver fibrosis and improved liver function. Wang et al. induced rat hepatic fibrosis via intraperitoneal injections of CCl_4_ twice a week for 12 weeks, where the initial dose of CCl_4_ (diluted 1:1 in olive oil) was 5 mL/kg and each subsequent dose was 3 mL/kg (diluted 1:1 in olive oil) (Wang et al., 2012b). Yamaza et al. (2015) used a mixture of CCl_4_ (0.5 ml/kg body weight) and olive oil (1:4 *v*/*v*) and intraperitoneally injected it into C57BL/6J mice (male, 8 weeks old) twice a week. Regarding the route of CCl_4_ administration and dosage, we used the intraperitoneal injection method for CCl_4_ administration, but the fatality rate was more than 90% in our preliminary experiment. Therefore, in the present study, liver fibrosis was induced in Kunming mice by CCl_4_-gavage every three days for eight weeks, and the final survival rate was 32%. This low survival rate may be due to that the Kunming mice are more sensitive to o CCl_4_ intraperitoneal injectioncompared to other mouse strains. A total 10^6^ EGFP-labeled MSCs were transplanted via the caudal vein and survived at least for one month before euthanization in this study. One month after MSCs infusion, none of the mice were dead in both CCl_4_ group (*n* = 3) and CCl_4_ + MSCs group (*n* = 4). This may be because during the 8-week administration of CCl, only the mice (32%) that were less sensitive to the toxicity of CCl_4_ survived. When the CCl_4_ administration ceased, there were no further damages to the mouse livers. Therefore, the mice survived even without transplantation of MSCs. However, compared to the mice that received the infusion of MSCs, the liver fibrosis was not ameliorated. Our results showed that the transplantation of rhesus macaque bone marrow-derived MSCs obviously improved the liver function of mice with liver fibrosis evaluated by the blood serum levels of ALT, AST, ALB and TP. Moreover, the liver fibrosis progression and hepatocyte necrosis were decreased, which was justified by the pathological evaluation. In addition, the gene expression of the iconic molecules associated with liver fibrosis (AFP, α-SMA, ALB, TNF-β) in mouse liver tissues was examined. The expression of these genes, the typical molecular markers of liver fibrosis (α-SMA and), hepatocyte functional marker (ALB), and liver injury marker (AFP and TNF-β) (Tanimoto et al., 2013), was obviously changed after MSCs transplantation. Our results indicated that heterogenic transplantation of EGFP-labeled rhesus macaque bone marrow-derived MSCs effectively improved the liver fibrosis syndrome of CCl_4_-administered mice in terms of their pathology as well as the gene expression levels of serum and liver fibrosis-associated genes. Besides this, we also detected the apoptotic marker (caspase-3) and proliferous factor (Ki-67) in the liver of CCl_4_-induced liver fibrosis mice after MSC infusion. The results showed that apoptosis decreased after heterogenic transplantation of MSCs. In contrast, the hepatocyte proliferation was not affected by MSCs infusion. Our results indicate that, instead of facilitating hepatocyte proliferation, MSCs improve liver fibrosis via their anti-apoptosis effect.

Similar to our results, the transplantation of human MSCs in rodent liver fibrosis models appeared to decrease liver fibrosis and improve liver function. However, with regard to whether the therapeutic transplanted MCSs differentiated into hepatocytes or secreted trophic factors to improve liver functions was not illustrated (Aurich et al., 2007; Kubo et al., 2015; Yamaza et al., 2015). A few studies have reported that heterogenic transplanted MSCs can be found in the liver tissues of mouse receptors (Chang et al., 2009; Kuo et al., 2008; Rabani et al., 2010). Recently, a study revealed that transplantation of human bone marrow MSCs successfully rescued fulminant pigs with hepatic failure, and only about 4.5% of the human MSCs integrated and differentiated into hepatocytes in the pig liver tissue. The results of this study also indicated that the implanted human bone marrow MSCs altered the cytokine responses of the pig to liver injury through paracrine effects (Shi et al., 2016). Chen et al. (2017) found that human menstrual blood-derived MSCs markedly ameliorated the liver fibrosis of the CCl_4_-induced mouse model, but only a few GFP-labeled MSCs differentiated into functional hepatocyte-like cells. Meanwhile, they illuminated that the proliferation of hepatic stellate cells was suppressed by MSCs through the secretion of monocyte chemoattractant protein-1, interleukin-6, hepatocyte growth factor, growth-related oncogene, interleukin-8, and osteoprotegerin using transwell coculturing experiments. Zhang et al. (2012a) used human umbilical cord-derived MSCs, which improved the CCl_4_-induced liver failure mouse models by decreasing the levels of inflammatory cytokines such as interleukin (IL)-1 β, tumor necrosis factor (TNF)-α, IL-6, and IL-10 *in vivo*, and they further found that the cell viability and ALB secretion ability were significantly increased through the coculture of MSCs and injured mouse hepatocytes. The study declared that the paracrine effects of MSCs stimulate endogenous liver regeneration rather than hepatic differentiation, which compensated for liver function. These results indicated that MSCs may rescue liver dysfunction and liver fibrosis mainly through paracrine effects since only a small number of transplanted MSCs homed and engrafted in the liver receptors. Similar to these previous studies, only a small number of heterogenic EGFP-labeled MSCs (1.8%) were detected in mouse liver tissue after 4 weeks of transplantation in our study. Usually, MSCs are administrated via intravascular injection and intraperitoneal injection and then spread to most organs like liver, heart, kidney and intestine and exert effects in the animal’s body. The majority of infused MSCs tend to form aggregate in the lungs than other organs, and the infused MSCs gradually disappear 7-14 days post injection. In this study, we observed that a limited number of MSCs homed into the liver and survived up to 30 days, which is similar to previous reports (Kurtz, 2008; Bazhanov et al., 2016). Though MSCs have low immunogenicity but they can still be easily eliminated by the immune system after exerting efficacy in a short period (Wang et al., 2014; Zheng, Wang & Xu, 2016), which may explain the fate of the transplanted cells. Then, the functional characteristics of MSCs including lymphocyte proliferation inhibition and excretive cytokine ability were detected *in vitro*, and our results indicated that the paracrine effects of MSCs may play an important role in the improvement of liver fibrosis.

## Conclusions

Heterogenic MSCs ameliorate liver fibrosis in mice, and a small number of MSCs homed and engrafted in mouse liver tissues. The MSCs respond to interferon-γ stimulation and have the ability to inhibit lymphocyte proliferation *in vitro*. Our results showed that transplantation with heterogenic MSCs derived from monkey bone marrow can treat liver fibrosis in a mouse model and that the paracrine effects of MSCs may play an important role in the improvement of liver fibrosis. However, the detailed therapeutic mechanism still needs to be intensively studied in the future.

## Supplemental Information

10.7717/peerj.4336/supp-1 Supplemental Information 1 The raw data of Fig. 5A Weak electrophoresis bands of EGFP presented in the mouse liver tissue infused with labeled MSCs (lanes 2 and 3), where lane 1 is the negative control and lane 4 is the positive control.

10.7717/peerj.4336/supp-2 Supplemental Information 2 The raw data of Fig. 7 The GAPDH expression of MSCs were treated with IFNγ h.

10.7717/peerj.4336/supp-3 Supplemental Information 3 The raw data of Figs. 7–2 The chemokine and migration-related genes expression of MSCs were treated with IFNγ.

## Additional Information and Declarations

### Data Availability

The following information was supplied regarding data availability: The raw data for Figures 5 and 7 are provided as Supplemental File.
