# Supplementary figures and images for: Heterogenic transplantation of bone marrow-derived rhesus macaque mesenchymal stem cells ameliorates liver fibrosis induced by carbon tetrachloride in mouse
Source: PeerJ. 2018 Feb 12;6:e4336. doi: 10.7717/peerj.4336 (PMC5813592; doi:10.7717/peerj.4336)

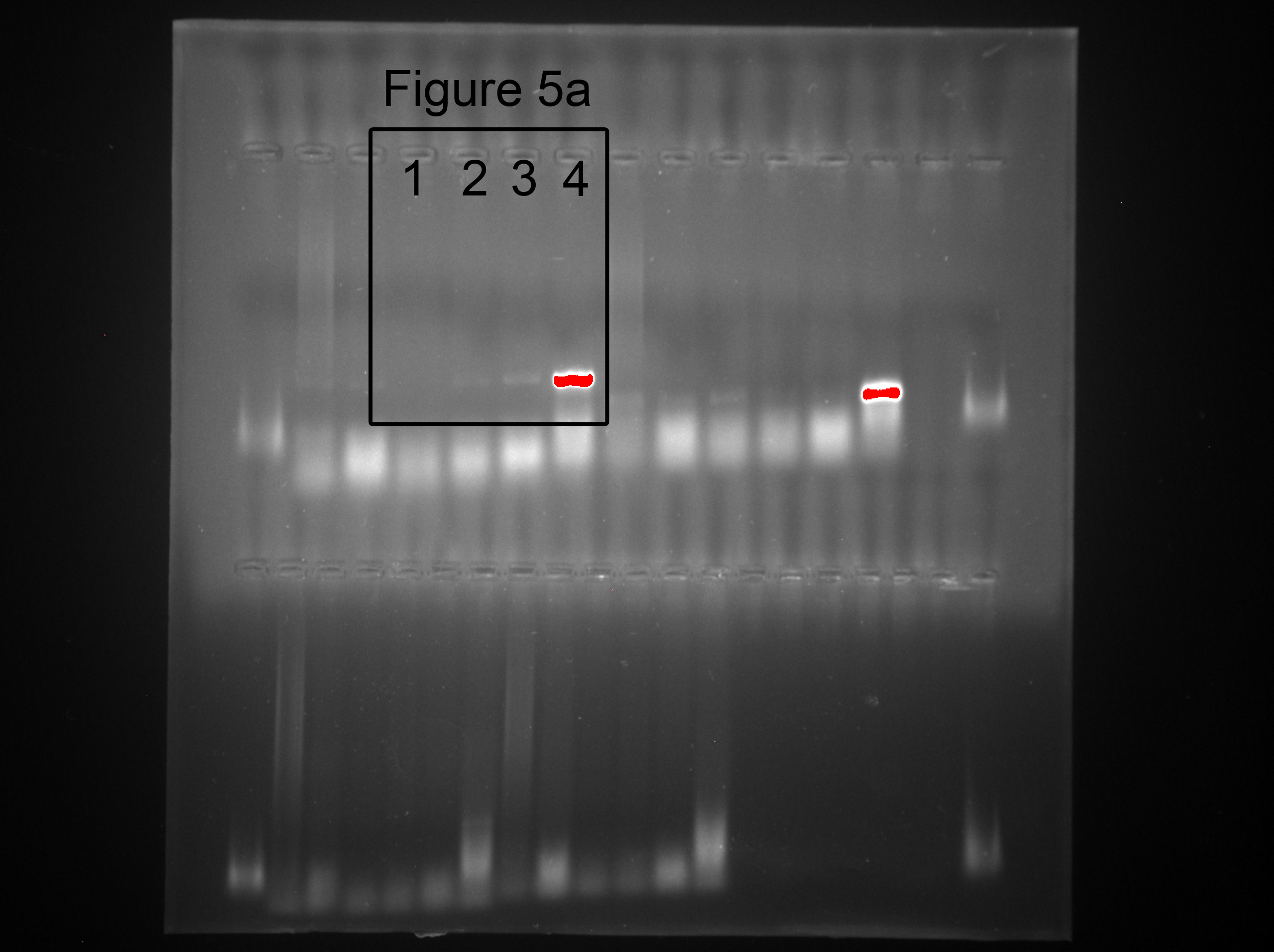

Supplement: Supplemental Information 1 — Weak electrophoresis bands of EGFP presented in the mouse liver tissue infused with labeled MSCs (lanes 2 and 3), where lane 1 is the negative control and lane 4 is the positive control. [file peerj-06-4336-s001.jpg]

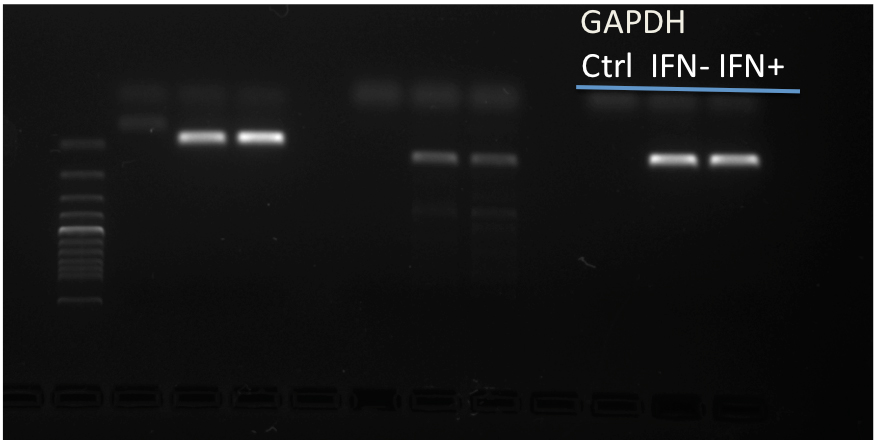

Supplement: Supplemental Information 2 — The GAPDH expression of MSCs were treated with IFNγ h. [file peerj-06-4336-s002.jpg]

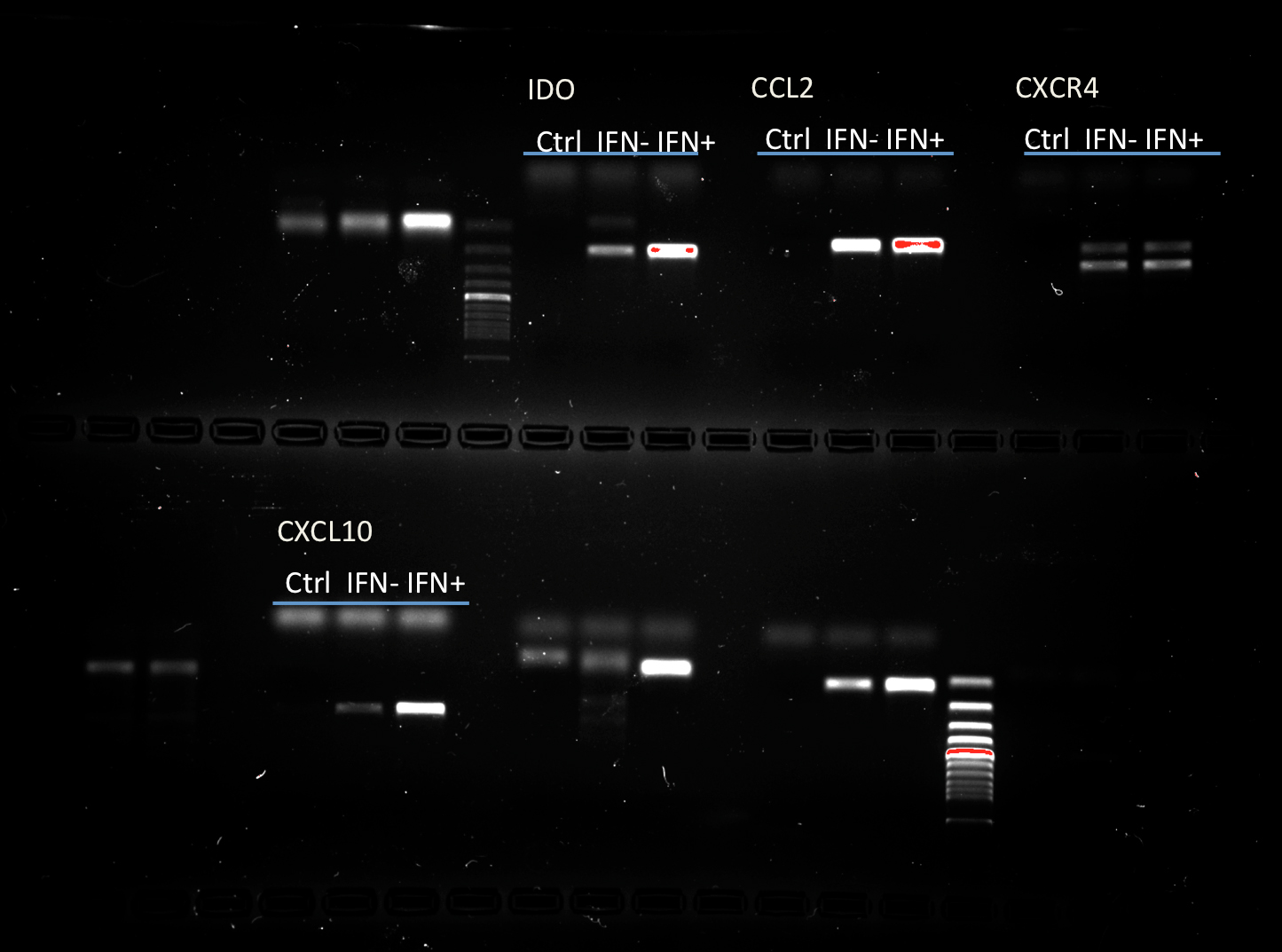

Supplement: Supplemental Information 3 — The chemokine and migration-related genes expression of MSCs were treated with IFNγ. [file peerj-06-4336-s003.jpg]
